# Framework for qualifying exoskeletons as adaptive support technology

**DOI:** 10.3389/frobt.2022.951382

**Published:** 2022-12-21

**Authors:** Oliver Ott, Lennart Ralfs, Robert Weidner

**Affiliations:** ^1^ Chair of Production Technology, Institute of Mechatronics, University of Innsbruck, Innsbruck, Austria; ^2^ Laboratory of Manufacturing Technology, Helmut Schmidt University, Hamburg, Germany

**Keywords:** exoskeleton, support technologies, smart device, human-machine interaction, support characteristic, adaptivity, system behavior, framework

## Abstract

The fifth industrial revolution and the accompanying influences of digitalization are presenting enterprises with significant challenges. Regardless of the trend, however, humans will remain a central resource in future factories and will continue to be required to perform manual tasks. Against the backdrop of, e.g., societal and demographic changes and skills shortage, future-oriented support technologies such as exoskeletons represent a promising opportunity to support workers. Accordingly, the increasing interconnection of human operators, devices, and the environment, especially in human-centered work processes, requires improved human-machine interaction and further qualification of support systems to smart devices. In order to meet these requirements and enable exoskeletons as a future-proof technology, this article presents a framework for the future-oriented qualification of exoskeletons, which reveals potential in terms of user-individual and context-dependent adaptivity of support systems. In this context, a framework has been developed, allowing different support situations to be classified based on elementary functions. Using these support function dependencies and characteristics, it becomes possible to describe adaptive system behavior for human-centered support systems such as exoskeletons as a central aspect. For practical illustration, it is shown for an exemplary active exoskeleton using the example of user-individuality and context-specificity how the support characteristics of exoskeletons in the form of different support characteristics can bring about a purposeful and needs-based application for users and can contribute valuably to design future workplaces.

## 1 Introduction

Over the past decade, the focus in industrial factories has evolved significantly due to the influences of the fifth industrial revolution. Trends such as digitalization (Di Pasquale et al., 2021; Mark et al., 2021; Mark et al., 2022) and automation (Wang, 2018; Casla et al., 2019; Di Pasquale et al., 2021; Mark et al., 2022) have shifted paradigms towards a smart and connected factory (Mark et al., 2021), resulting in complex and dynamic work environments (Mark et al., 2021; Mark et al., 2022) with intensified human-machine collaboration (Casla et al., 2019) and interaction (Weidner et al., 2016; Weidner et al., 2017).

Regardless of the increased importance of digitalization and automation in this technocratic perspective of the fifth industrial revolution, human operators with their individual skills and capabilities will not be excluded from production (Al-Ani, 2017; Di Pasquale et al., 2021; Mark et al., 2021), but continue to be an integral part and indispensable factor (May et al., 2015; Casla et al., 2019; Mark et al., 2021) in the center of attention (May et al., 2015; Romero et al., 2016; Casla et al., 2019; Mark et al., 2022) of future industrial operations. However, it is expected that the operators’ tasks will change fundamentally (Romero et al., 2016; Casla et al., 2019) due to changing job profiles and requirements. This issue becomes even more relevant against the backdrop of, e.g., advancing social and demographic change (European Agency for Safety Health at Work et al., 2020; Hoffmann et al., 2021), through which companies are confronted with limited access to skilled personnel as well as aging and fluctuating compositions of the workforce (Casla et al., 2019; Sgarbossa et al., 2020; Di Pasquale et al., 2021). In order to keep pace with the developing role of human operators in factories, a complementary transformation of workplaces and processes is required. In this manner, workstations are being further developed into human-centered and new sustainable workplaces with according work processes (May et al., 2015; Casla et al., 2019). Nevertheless, industrial work processes are still characterized by a considerable share of manual activities, e.g., carrying heavy loads in logistics or performing repetitive assembly tasks in awkward postures in production, whose demands and stresses on the operator differ depending on the activity. As a result, the operators are both physically and psychologically strained and, first and foremost, exposed to the risk of suffering from musculoskeletal disorders (Roquelaure, 2018; European Agency for Safety Health at Work et al., 2020). More specifically, musculoskeletal disorders account for around one-quarter of the days of incapacity to work in industry (Bundesministerium für Arbeit und Soziales, 2021).

Accordingly, a trend emerges toward physically and cognitively supporting operators with technologies (Mark et al., 2021), which are, among other effects, capable of preserving their healthy conditions (Sgarbossa et al., 2020) if applied appropriately and target-oriented. In an industrial context, support technologies are technical devices that support but do not override or replace operators in performing industrial tasks (Weidner et al., 2013; Mark et al., 2022). They make it possible to simplify the interaction between humans and technology, thereby strengthening the physical and cognitive skills of operators (Mark et al., 2022) and helping reduce the perceived complexity of work processes (Hinrichsen and Bornewasser, 2019). In scientific literature, a fundamental distinction is made between sensorial (e.g., motion tracker, eye tracking system), physical (e.g., exoskeletons, robots, lifting aids), and cognitive (e.g., Augmented Reality (AR), Virtual Reality (VR), projection systems, voice control) support systems (Romero et al., 2016; Mark et al., 2021). Another classification only distinguishes between physical and cognitive support (Galaske et al., 2019). Compared to the previous approach, the sensory approach of cognitive support is subordinated to perceptive support. Just like AR, VR, and smart aid tools, exoskeletons are becoming increasingly important and of growing interest for the physical support of operators in industrial workplaces (Hoffmann et al., 2021; Mark et al., 2021; Mark et al., 2022). While exoskeletons were initially used in rehabilitation and the military, they nowadays are of increasing interest in supporting employees in industry and the consumer sector (Bogue, 2018). Accordingly, exoskeletons for industrial applications are the subject of this article and are intended to contribute to the prevention of musculoskeletal disorders. Corresponding exoskeletons are systems worn on the human body to provide physical support to stressed body regions (Looze et al., 2016; Fox et al., 2020; Weidner and Hoffmann, 2020). Depending on the morphology, functional design, and applied principle of action, different forms of support such as facilitating, stabilizing, or adding movements can be enabled to varying degrees (Weidner and Karafillidis, 2018).

**FIGURE 1:**
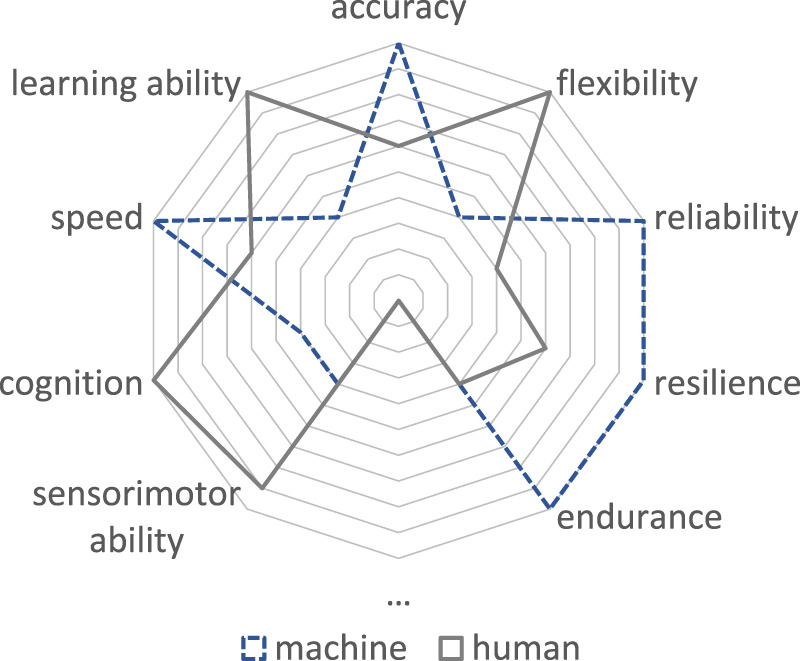
Qualitative representation of individual skills and abilities of human operators and technical systems.

Figure 1 qualitatively illustrates the different expressions of skills and capabilities of humans and technical systems. The advantages of humans, e.g., lie in their physical strength and cognitive abilities (Mark et al., 2022). Similarly, compared to technical systems, sensorimotor ability, learning ability, and flexibility (Di Pasquale et al., 2021) are more pronounced. On the contrary, technical systems offer the advantage of high processing speeds combined with high (repeatable) accuracy, reliability, and endurance. On the one hand, the advantages of both resources can be combined through intelligent hybridization or coupling of people and technology. Thus, the respective strengths can complement each other so that the symbiotic relationship can increase their skills, abilities, and capabilities and the overall quality of the work (Casla et al., 2019). On the other hand, it also becomes possible to compensate for the weaknesses and deficits of human operators (Mark et al., 2021; Mark et al., 2022) by using the partly contrasting but at the same time complementary capabilities of technical support systems.

In the past, workers had to be able to adapt to machine conditions (Casla et al., 2019). In contrast, to meet the requirements of the dynamic and complex working environment with its diverse work tasks and high product individualization, highly flexible and adaptable operators are needed in the future factory (Longo et al., 2017; Merkel et al., 2017; Mark et al., 2021). Only this way will it be possible to achieve the necessary flexibility and agility in the future. Since there is a close dependency between the perceived complexity, the situation-specific functionality of the support technology, and the operator’s skills, these aspects must be dynamically adjusted to each other (Hinrichsen and Bornewasser, 2019). Support systems such as exoskeletons are well suited to cope with the increasing complexity of manual production (Merkel et al., 2017). When exoskeletons are intended to support humans, the technology and its interaction with operators (Di Pasquale et al., 2021) must also be capable of adapting and coping with changing conditions. Thus, to use exoskeletons purposefully and appropriately for support, adaptive systems are required with regard to the respective user and the context of the application, e.g., the level of support (Casla et al., 2019). Concerning exoskeletons, this means that a process-optimized interaction of human and system needs to take place in flexible production (Dahmen and Constantinescu, 2020). Consequently, system adaptivity is the key to reacting in the best possible way to given framework conditions such as workplace dynamics, product versatility, or process variation.

Up to now, the way to further qualify exoskeletons into an adaptable support technology has neither been sufficiently investigated nor generically addressed by scientific articles yet. Accordingly, a superordinate and standardized tool lacks describing the characteristics of support technologies such as exoskeletons, creating the basis for adapting technology to the user and the context. This article presents a central framework for qualifying exoskeletons as adaptive support technology regarding users and tasks by addressing and tackling the above challenges as well as the following three research questions (RQ):

RQ1: What forms of adaptivity are relevant to consider in connection with exoskeletons while also being applicable to other physical support systems?
RQ2: What are the crucial levels and elements of an adaptivity framework for describing and determining the adaptive support behavior of exoskeletons?
RQ3: How can the framework be practically applied to an exemplary exoskeleton and scenario?

By answering these questions, the framework helps classify different support situations with which various functional dependencies and characteristics can be combined. The framework is designed for the current state of development of available exoskeletons. It is therefore emphasized that not all exoskeletons have the same functionality and differ in their adaptation options. Besides, the framework is mainly developed for the application range of exoskeletons supporting workers in industrial workplaces.

## 2 Fundamentals for developing the framework

Adaptivity, in general, describes the adaptation to a situation that has changed within a time offset from the initial situation (Inagaki, 2001). In terms of human-machine interaction, this refers to the necessary adaptation of the system and its support behavior to the context. The context is raised by the user with his experience, skills, and qualifications, performing a specific task in its complexity and deterministic sequence with the appropriate tool in a particular work environment and under the given external influences (Casla et al., 2019; Hoffmann et al., 2021; Ralfs et al., 2022) at a specific moment of the scenario, the support situation. The support of the exoskeleton can thereby be described as the relationship between the angular range within the motion space of the supported body joint, and the corresponding applied force or generated torque. Adapted to a specific support context, this results in a support function with an individual support character due to the profile with its level of torque and gradient over the angles, in the form of a force-angle curve or torque-angle curve (in the following generally referred to as support curve). The unique support characteristic of an exoskeleton thus results from the entirety of available support curves, consisting of the sum of support characters. The properties of an exoskeleton are reflected in the outwardly visible system behavior.

### 2.1 Characteristics of exoskeletons

Exoskeletons can be divided into three main categories regarding their energy use. Systems that can store the energy of a body’s movement in one direction in order to return it in the opposite direction in the form of support are referred to as passive systems. This category also includes systems that can dissipate or harvest the energy of the movement and return it to the person in a modified kinematic form. Passive systems often incorporate elastic elements such as springs or rubber bands for their actuation (Manna and Dubey, 2018). Alongside these are active systems that use an external energy source, for example, to adjust the level and course of support during movement to match the user’s activity. Sorted in descending order of frequency, electric motors (e.g., brushed or brushless DC motors), pneumatic actuators (e.g., pneumatic artificial muscles), and to some extent, even hydraulic actuators are used (Tiboni et al., 2022). In general, active systems can adjust the support characteristics in the application through active control and the implemented sensor technology. Depending on the controlled variable, dynamic sensors [e.g., pressure, torque, force sensors, an inertial measurement unit (IMU)], encoders, and electromyographic (EMG) sensors are used to measure physical quantities such as forces, torques, acceleration, position, displacement, and muscle activities (Tiboni et al., 2022). The hybrid approach of semi-active systems represents another category. These systems use an external energy source to adapt the inherently passive support characteristics to the changing demands of varying activities. This behavior can be achieved by using compliant actuators (Manna and Dubey, 2018) such as series elastic actuators (Ham et al., 2009).

There are various approaches and starting points for designing or qualifying both user- and situation-dependent exoskeletons. The connecting points differ depending on the degree of the desired adaptability or adaptivity and the resulting design of the exoskeleton. This means that adaptation takes place on the one hand during the development phase through the qualification of the system by the developer (and experts) as well as on the other hand through adaptation in the application by the user or the system itself. The range of possibilities for adaptation differs and, in turn, is conditioned by the functionality, the constructive form of the exoskeleton, and the choice of actuation.

### 2.2 Adaptation possibilities

**FIGURE 2:**
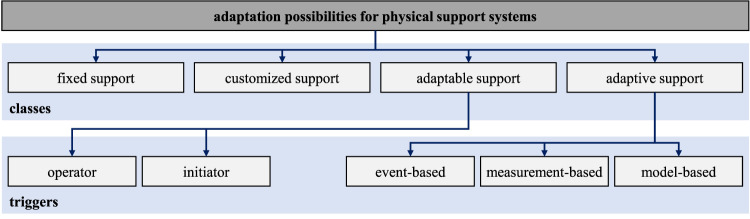
Classification of adaptation possibilities for physical support systems by means of different classes (Wandke, 2005) and triggers (Burggräf et al., 2021)

Accordingly, the constructive design of the exoskeleton and the considered actuation type intensely condition the possibilities of adapting the exoskeleton in hardware and functionality to the user and the support behavior. Therefore, different forms of adaptation are considered in the literature, especially concerning support systems (Figure 2). At this point, it should be noted that in the context of support technologies, the term assistance is often used and widespread. In this article, however, the term support is used consistently.

#### 2.2.1 Fixed support

As early as the development phase of an exoskeleton, adaptation takes place, for instance, by implementing direction-dependent stiffnesses in the constructive design or considering kinematic degrees of freedom for the best possible mapping of the human range of motion. The provided actuator technology will be selected to support the intended activity concerning the physiological requirements of the user. If this development takes place with regard to a fixed support characteristic for all planned tasks, it is called fixed support (Wandke, 2005). This support is thus context-independent and is accordingly provided to the same extent and independently for each user without considering the current situation. A subsequent adjustment is not foreseen and, therefore, not possible in the application. Consequently, the support characteristics must be designed, if possible, to support a wide variety of different tasks and provide the necessary flexibility in the application without hindering the user while performing secondary activities, e.g., due to limited range of motion or disruptive counterforces in movement phases. However, this constant and thus deterministic behavior of the system bears the risk that the system does not provide support to the expected extent in parts of a task or increasingly interferes with the user in the execution that was not or not sufficiently considered in the development phase (Hinrichsen and Bornewasser, 2019).

#### 2.2.2 Customized support

This means that the exoskeleton must be developed and designed with the application and users in mind. An analysis of the later users and the application to be supported is thus inevitable for the development process. This goal-oriented development and the corresponding focus on a use case or a context leads to customized support. As with fixed support, the customization of the system already takes place in the development phase and thus inevitably without the possibility of taking into account changing environmental influences, deviating tasks, or users.

#### 2.2.3 Adaptable support

Consequently, the possibility for later adaptations in the application can already be provided in the development phase to create the necessary flexibility required to react to, for example, unforeseen variations in the task or varying users. This adaptation is then carried out by the user himself (operator) or, e.g., by an appropriately qualified expert (initiator) (Burggräf et al., 2021). In addition to the adaptability of the exoskeleton in its kinematic structure and the intended degrees of freedom to the user’s anthropometry, the exoskeleton’s physical interfaces can also be adapted to the user to improve the interaction with the exoskeleton. To realize the adaptability of the support, it may be possible, for example, to vary stiffnesses, exchange actuators, or modify the constructive shape of the mechanical support structures. This manual modification of the structure offers at least some possibilities of adaptability of the system for selected activities and users. The implemented control for active or semi-active systems further allows the specification of different support curves adapted to, e.g., the intended activities, specific tools, corresponding movements, individual preferences, or physiological requirements. As a totality of selectable support curves and adjustable characters, this support characteristic enables the user- and situation-dependent adaptability of the exoskeleton in the application by the user or an expert (Burggräf et al., 2021).

#### 2.2.4 Adaptive support

However, if the exoskeleton’s adaptation is made without the need for manual intervention (Burggräf et al., 2021), the support is considered adaptive (Wandke, 2005). This system adaptation can basically be triggered by 1) a specific measurement result, 2) an occurring event, or 3) an underlying model (Inagaki, 2001):

1) The sensor technology integrated within active and semi-active systems for the implementation of the control can be used to collect data related to the performed task and use them to adapt the support or to design systems depending on the general scenario. These data can be related, for example, to exceeding and falling below a threshold value such as the current level of support, certain accelerations in the movement, or to data of production processes such as the processing time or the error rate (Burggräf et al., 2021).
2) The interpretation of the recorded data can also inform the system about the occurrence of an event and thus act as a trigger. This event could be the occurrence of pre-defined scenarios (Burggräf et al., 2021). For example, depending on the design of the sensors, the system can enable connectivity and thus communicate with the immediate or work environment, such as tools used. If, for example, the system registers a change of workstation or tool, and thus a change in the task performed, the system can automatically adapt to the requirements of the new activity and prepare the respective support, for example, in the form of an adapted support curve.
3) The data recorded by the system in the task can also be the data basis and support the qualification of, e.g., mathematical models. Besides using, e.g., inference models, mathematical and resource models can be used to make predictions and estimates about current states and future movement patterns (Romero et al., 2015; Burggräf et al., 2021). Based on these calculations, the system can also estimate the support needed at the next step concerning the task performed and the corresponding work environment. Depending on the quality of the model, it may then even be possible to provide targeted support for activities not defined in advance.

Regardless of the final trigger, the system’s adaptability is based on measured data and the work context perceived by sensors, which the system uses to adjust the support parameters (Wandke, 2005).

### 2.3 Implications for the framework

**FIGURE 3:**
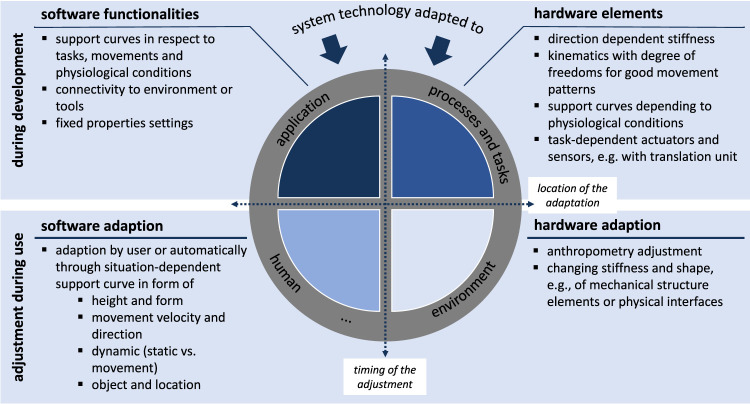
Possibilities of adaptation during the development phase and in application in hardware and software in exoskeletons.

It can be summarized that different stakeholders can be supported depending on whether the adaptation occurs during the development or the use of an exoskeleton. If it is to help realize a relevant support characteristic during development specifically tailored to the respective context, it makes sense to store presets and support curves on the exoskeleton for different scenarios. In this respect, adaptation can be based on software functionalities or hardware elements. Concerning the software, this can be, for example, the consideration of support curves, the connectivity with the environment, or the property settings. An influence on the hardware can be, among others, the degrees of freedom of the exoskeleton or the sensors and actuators installed in the system. If, however, the focus lies on the application by the worker, the focus may be less on the retrieval of stored support curves but rather on the feasibility of specific support characteristics of the entire system by means of adaptive and adaptable behavior. In this phase, however, the focus of the adaptation is on the situation-dependent force curve (software), the change of structural elements, or the adjustment of the system to the anthropometry. Figure 3 summarizes the relationship between the possibilities for adaptation in the development phase and the later application by the user or the system itself and the adaptable components or elements of such systems (in software or hardware).

## 3 Method for qualifying exoskeletons

Due to the unaffected and increasing complexity of human beings and activities, respectively, the system’s adaptation to the user and the application context will continue to play a crucial role. Accordingly, different resources and actors in the context of exoskeleton use must be considered in the development of the framework, which aims to provide a fundamental systematic for describing different support functions, helping to classify support situations in industrial scenarios, and identifying ways for adaptable and also adaptive system behavior concerning support characteristics. In order to tackle this issue, a central framework for achieving adaptive system behavior with its logical links and interrelationships between different influencing factors is developed and, in the following, explained in a superordinate manner. It addresses how adaptable and adaptive system behavior can be achieved for activity profiles with their task-specific properties. In this context, it is crucial at which point in time the exoskeleton should support the user and how the system should react in certain situations. The framework primarily helps describe possibilities for software adaptation during the use of exoskeletons and during their development phase. Accordingly, there is a requirement to create a universally applicable and comprehensive framework to describe the support characteristics for exoskeletons.

### 3.1 Presentation of the general framework

**FIGURE 4:**
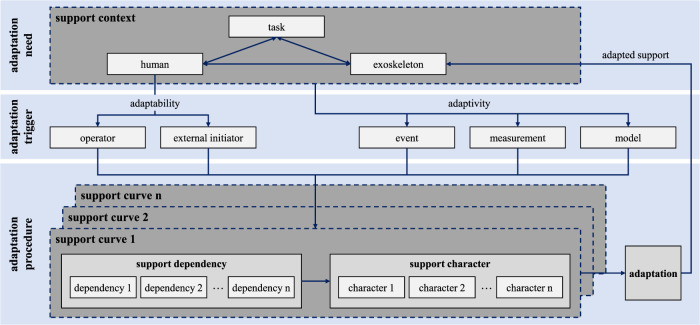
Framework for achieving adaptive system behavior of exoskeletons.

In order to be able to map the relevant aspects of adaptation in the framework, it is essential to consider multiple dimensions of influence. Thus, the framework is fundamentally divided into three sequential parts: the need to adapt the support in the first step, the adaptation trigger in the second step, and finally, the adaptation procedure describing the support characteristics of exoskeletons. The fundamental need for adaptation arises from the support context and the interaction of the human, exoskeleton, and activity. Accordingly, the support situation must be viewed holistically as the interaction of people, exoskeleton technology, and activity (Weidner et al., 2013; Di Pasquale et al., 2021; Hoffmann et al., 2021; Ralfs et al., 2022). The interactions may require a corresponding adaptation of the support characteristics. In a second step, these dimensions can cause a need for adaptation and trigger it. Thus, on the one hand, the interaction of the user, exoskeleton, and activity, and on the other hand, specifically the user, can cause the adaptation (see specific description of triggers in Section 2). Accordingly, depending on the scenario, the adaptation can be caused either by an event, a measurement, or a model (in the case of adaptivity) or by the respective user or an external initiator (in the case of adaptability). Regardless of the trigger, the next and central step is to adapt the support curves of the exoskeleton. Subsequently, the exact characteristics can be specified in more detail (e.g., level and instantaneous gradient of the support depending on the angular position). A possible switch of the support curve then adapts the support behavior for the respective context. The result of the adaptation procedure can thereby also be that the support curve does not have to change for the respective context but can also continue to be suitable. Figure 4 illustrates and summarizes the described relationships.

### 3.2 Composition of support curves

It is advantageous for a demand-oriented use of exoskeletons if the support curves are pre-defined differently to match the heterogeneous requirements of the later work context. Accordingly, the support curves of the exoskeleton should be designed according to whether the stabilization of postures (e.g., of the arms when performing assembly activities above head height), facilitation (e.g., during dynamic movements or by reducing load peaks), or enhancement (e.g., strengthening power and endurance of users) of movements is in the foreground. Thus, depending on the angular positions in which the provision of support by the exoskeleton is required, higher support is not necessarily to be evaluated as better. This point is particularly relevant because, for example, the generation of torque on the human body can also have a restrictive effect when performing secondary activities (such as climbing stairs or walking) or in specific forced postures. However, an adaptive system behavior for exoskeletons makes it possible to take these framework conditions into account and initiate a suitable level of support. Basically, the adaptivity of the exoskeleton is essential to create a good synergy with people, as a higher ability to adapt is to be considered better.

**FIGURE 5:**
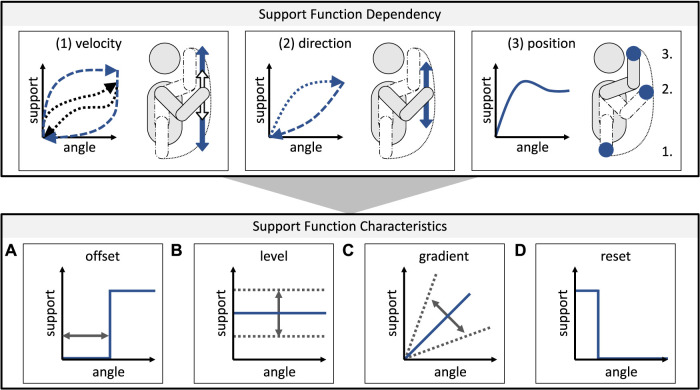
Overview of building blocks for describing support curves of exoskeletons for the example of the elevation movement of the arm when performing assembly activities above head level.

Since the basic framework has been presented, the composition of the support curves will be examined in more detail. Thereby, the description of the support curves can generally be based on fundamental dependencies (referred to as 1–3) and specifying characters (referred to as a-d), as illustrated in Figure 5.

#### 3.2.1 Support dependencies

In the first step, an exoskeleton’s general behavior is defined. From a physical point of view, integrating the accelerations for different angles allows conclusions to be drawn about the respective 1) velocity and its level in terms of the absolute value, 2) direction, and 3) current position. As a result, the support provided by exoskeletons can be described as a relationship between the body pose, the direction or absolute velocity of movement, and the force or torque applied to the human body in the joint. Besides the possible additional use of tools, these three variables are suitable for the basic description of the support curves of the exoskeleton. Concerning the use of exoskeletons in practice, this means that the dependencies are of different importance depending on the users’ anthropometrics and the work context (e.g., with regard to dynamic or static tasks or the occurring variance in the performance of tasks). Accordingly, these support curves have a decisive influence on the suitability of exoskeletons for user-individual and context-dependent support. By doing so, it is possible to adapt the support manually or to use pre-defined support curves which differ, e.g., in terms of level and gradient. These result in a specific function of the direction and velocity of motion (either dynamically to the movement, statically to a posture, or specifically to a location, a workstation, or a task). For static activities, for example, the respective pose and, correspondingly, the applied support in a narrow angular range is of decisive importance, while in dynamic activities with high variance, the direction and speed dependence in the support is central. Adaptive control of the exoskeleton technically accompanies direction and velocity dependence. At this point, the system’s adaptability for independent adaptation is already evident.

#### 3.2.2 Support characters

For the description of the support characters, four forms of expression are conceivable, based on which a detailed description of the support curve is possible for the entire motion space. These can be, on the one hand, 1) a time-delayed or angle-dependent offset or 2) a varying level of the support, and, on the other hand, 3) a varying gradient. In addition, 4) support reset at a defined point of time is conceivable. In terms of practical application, these four characters can further be explained:

1) An angle-dependent offset is relevant if support is only useful in a specific angular range and would even hinder other angular positions (e.g., during assembly tasks above head level with tool changes in between).
2) Varying the level of support provided can be beneficial in user-specific support since, e.g., different users may find the support comfortable to a non-uniform degree, or different work contexts may require different levels of support.
3) The gradient of the curve affects how much the exoskeleton’s support varies throughout the angle, e.g., in tasks with high variance, a high gradient may be conducive to meeting the varying support needs.
4) Furthermore, a reset of the support makes it possible, e.g., to stop the support when performing secondary activities.

### 3.3 General implications of the framework

The presented framework describes the relationship between the need for user- and context-dependent support, different adaptation triggers, and the resulting support applied by the exoskeleton. It can be used during system development or evaluation with correspondingly different examination purposes, e.g., to evaluate the adaptivity of existing exoskeletons, to assist their qualification to more adaptive support technologies, and to develop new adaptive exoskeletons. Accordingly, existing exoskeletons can be characterized in their adaptivity based on their respective adaptive triggers, and their support can be described using the methodology of support curves. Hereby, the methodology for describing the support curves with essential elements is similar to a construction kit, as the support at a certain angle is mapped by the composition of the support characters. The description of support as support curves created out of a construction kit is intended to enable experts, developers, and operators with the appropriate access to define support curves for particular tasks. A corresponding support curve results by extending it to the entire angle ranges. By describing the support as a function of the support height to a certain angle, the actuators’ inherent support of passive exoskeletons and the controlled support of active and semi-active systems can be expressed. Furthermore, the requirement of support for a given task can be described in the context of user- and situation-specific support. The exoskeleton can then be adaptively qualified or developed. In the case of passive exoskeletons, this qualification of further adaptivity could be achieved, for example, by adding requirement-specific replaceable springs with a spring characteristic curve that, in interaction with the exoskeleton kinematics, enables the defined and desired support curve for the selected task. For active exoskeletons, the corresponding support curves could be fed into the control system and adapted by the selected trigger during the task. In developing a new exoskeleton, this type of support requirement definition can guide the selection of actuators and the design of the kinematics. Overall, this approach of designing support curves for specific tasks and the possibility to implement multiple support curves within the exoskeleton, especially in active systems, reveals potentials of adaptive system behavior with a corresponding support characteristic using the adaptive triggers, which can support different work contexts and even physiological requirements of the user or task with different support curves situation-specifically.

## 4 Practical application example

The presented framework enables the qualification of exoskeletons as adaptable support technologies. As an illustration, the implementation of the framework will be demonstrated using the Lucy exoskeleton (Otten et al., 2018) as an example. Lucy is an active shoulder support exoskeleton designed to support activities at and above head level. The system uses pneumatic actuators and a sensor-based control which allows the support provided, in relation to the measured angle between the upper arm and the torso, to be adjusted by both the user and the system itself. The adaptation of the exoskeleton in its design and the support characteristics is both user- and situation-dependent.

### 4.1 Possibilities for adaptation of anthropometrics

**FIGURE 6:**
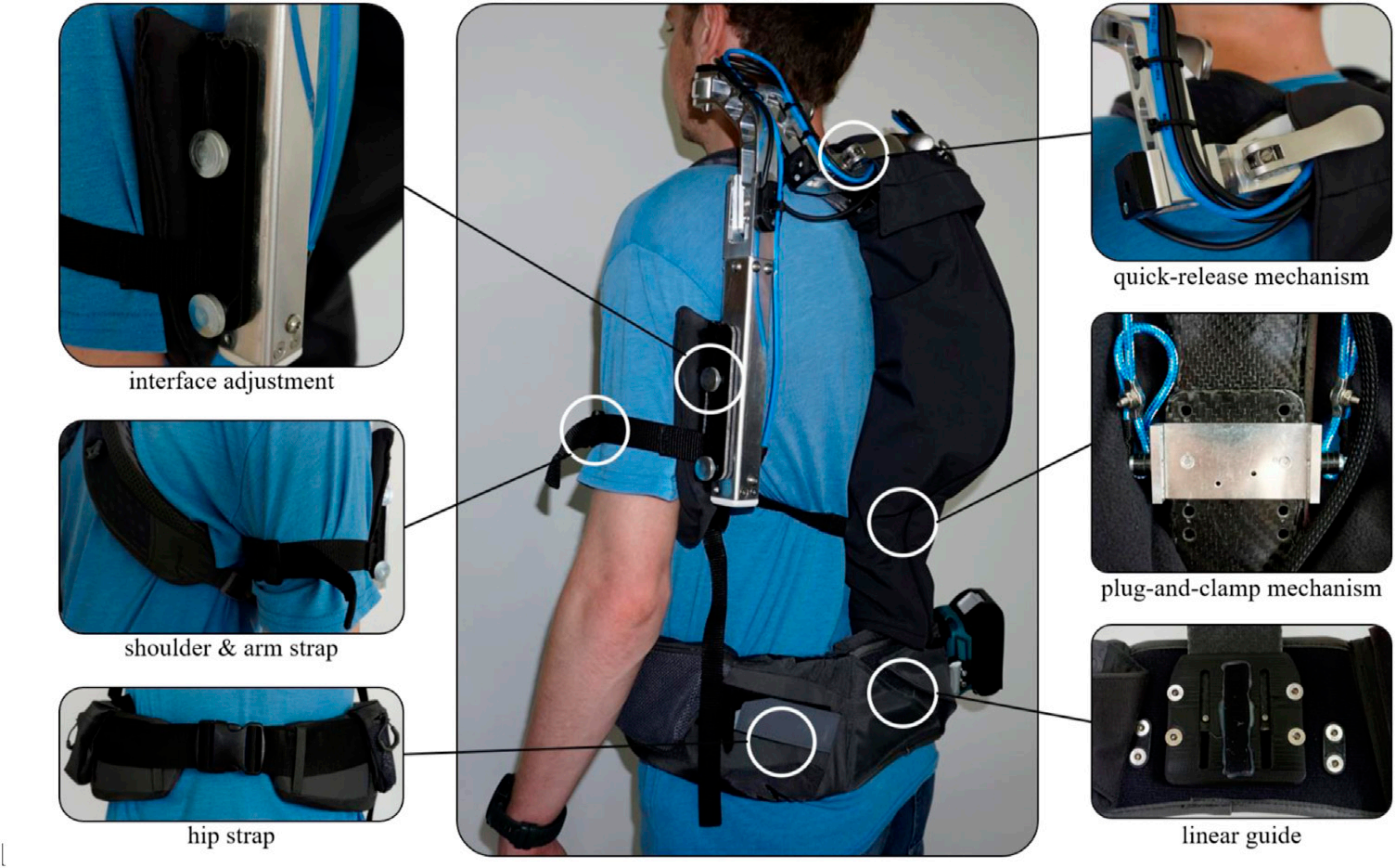
Adaptation possibilities to human anthropometry at the example of exoskeleton Lucy.

During the development phase, e.g., the structural design, constructive form, and the materials used allowed the exoskeleton to be adapted to the anthropometry of the user during use by the user himself or by an accompanying expert. For example, kinematics, intended to map the shoulder’s range of motion, can be manually adapted to the user’s dimensions. In realization, various quick-release systems help shift the system’s mechanical rotation axes via linear guides and thus adapt them to both the shoulder width and the biomechanical rotation axes of the shoulder. This anthropometric adaptation in the form of superposition plays a crucial role in mapping the human range of motion and preventing the development of friction zones in the interfaces between the system and the human due to relative displacements that could otherwise occur. In addition, Lucy can be adjusted in the height of the back element to the length of the user’s back using a simple plug-and-clamp system. This adaptation option allows the system to channel the support of the upper arms into the hips despite varying upper body lengths. The hip and shoulder straps implemented are adjustable in length and feature padding that facilitates their adaptability to individual body shapes. The large-scale half-shell elements of the interfaces are rigidly designed and also padded. They ensure good force transmission and yet reduce any pressure that may be applied at specific points. The interfaces can be adjusted along the upper arm, thus allowing the user to customize the force introduction into the exoskeleton depending on the length of the upper arm. An elastic and length-adjustable strap system at the interface ensures flexibility in adapting to the upper arm and secure support during movement. Using the exoskeleton Lucy as an example, Figure 6 shows the possibilities for adapting some design elements to the anthropometry of the user.

### 4.2 Possibilities for adaptation of support characteristics

The adaptation of the support characteristics plays a significant role for the user, especially during the application and when performing activities. Since Lucy is an active system, the adjustment is made with the help of an intervention in the control system. In turn, this intervention can be carried out by the user personally, an expert, or the system itself. In the simplest case, the user wants to adjust the level of support for a specific activity. This adaptation can be made manually and continuously via a rotary encoder on the built-in control element. Manual adjustment is beneficial because users find the level of support to be of varying comfort. The independent adjustment of the maximum support height simultaneously enables user-specific scaling of the pre-defined and activity-dependent support curve.

**FIGURE 7:**
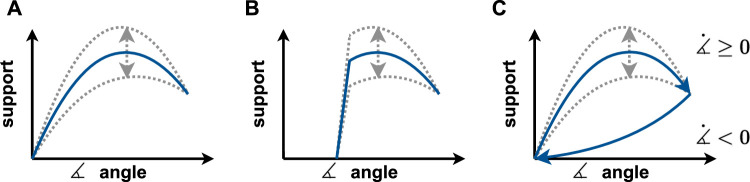
Three exemplary support curves for different scenarios: **(A)** angle-dependent but static force scalable in level; **(B)** static support for steady hold only at and above head level, scalable in height; **(C)** direction-dependent support curve for dynamic support, scalable in height.

In case of a change of the support context and thus other deviating demands for support, it may also be necessary to change the support character. For this purpose, the user can draw from a repertoire of preset support curves for different activities and tools. During the development phase, experts specified these support curves concerning the requirements of the intended tasks and integrated them for the situation-dependent control of the system. The selection of these curves can also be made through an intervention via the control element of the exoskeleton. However, the decisive factor in this adaptation is the suitability of the pre-defined support curves to the respective context. For this reason, the selection of different support curves (Figure 7) will be demonstrated using simplified examples. The presented examples vary according to their characteristics (e.g., dynamics) and variation parameters (e.g., tool use, work height, and spatial orientation) (Ralfs et al., 2021).

The support curves illustrate the relationship between the applied support and the angle between the upper arm and the torso. In a neutral angular position, the upper arm is located alongside the torso, and the angle increases as the arm is lifted. In addition to the support applied by the actuator, the illustrated curve also relates to the force transmission within the kinematics determined during the exoskeleton’s development phase. For example, the user could desire support for a specific activity across the entire angular range (Figure 7A). Depending on the current angle (location), the entire range of motion is supported with a precisely pre-defined level of support. This function can then be scaled in height by the user specifying its maximum force support via the control element. One such application is the handling of a cordless screwdriver, for example, which is to be picked up from the table, handled at or above head level, and then put down again. The tool weight is to be composed over the entire angular range by the exoskeleton, thus simplifying handling. However, if support is only desired in the range at or above head height, the force curve could provide a spatial offset for the corresponding lower angular range (Figure 7B). This force curve thus provides no support in small angular ranges but could still be manually scaled in the support force of the higher angles. A typical application is, for example, the manual handling of the wiring harness during underbody assembly in the automotive industry. The support is only needed during the overhead activity and can be perceived as disturbing during activities at lower angles, such as picking up screws. These illustrated support curves are in contrast to, for example, direction- or speed-dependent force curves. These curves still describe the relationship between the applied support force and the angle between the arm and torso but change in shape as a function of the direction or speed of the movement. As another example, a curve might support the elevation of the arm and the subsequent holding of a static posture of an assembly activity with an appropriate force (Figure 7C). However, if the system detects the desire to lower the arm by measuring a negative change in angle, the support force can be drastically reduced or inhibited, facilitating the lowering of the arm. The adaptation can be useful, for example, during the handling of a long-neck sander when sanding walls. The dynamic working method and the constant change of the working height are simplified by the direction-dependent support. This directional adaptation of the force curve is based on sensor signals from the exoskeleton and is thus adaptive.

The support characteristics can be adjusted depending on the recognition of 1) scenarios, 2) workstations and tools, and 3) mathematical models, among other things:

1) The independent adaptation of the exoskeleton based on sensor values can also take a higher-level position in the system’s control. For example, the orientation of the torso in space can already be determined with just one IMU in the back element of the exoskeleton. For example, if the user bends over, thus bringing the torso into a horizontal position, the support provided by the exoskeleton may interfere with or even hinder the activity at equal angles between the upper body and the upper arm. The adaptive system can automatically reduce or eliminate support for this pre-defined scenario.
2) Likewise, the exoskeleton, equipped with radio frequency identification (RFID) sensors, can sense its environment and thereby register, for example, the change of pre-defined scenarios, such as the change of a tool. This change can then be linked to force curves explicitly predetermined for the tool, enabling the system to select the defined curve to provide the appropriate support. The adaptation through the selection of the curves is thus no longer done by the user or an accompanying expert but rather by the tool and the system itself.
3) Based on the specification of the primary support characteristics of the exoskeleton in the form of force curves, the support can further be optimized by integrating models. Using a mathematical model to describe the relationship between the opening/closing times of the valve and changes in the pressure in the actuator allows an estimate to be made of the physical processes involved in generating the support. With knowledge of the current change in angle at run time due to integrated sensing, a predictive control can be set up that estimates the immediate next time steps and thus the support desired at that time. In combination with the description of the physical processes of force generation, the support can thus be provided in a targeted manner and at the point in time desired or even expected by the user, particularly in the case of dynamic activities.

In the application, the adaptation of the support of the exoskeleton Lucy takes place fluently. A possible sequence of the described adaptation possibilities is illustrated by the example of underbody assembly in automotive engineering and, thus, takes up the method and the examples raised above. The handling of a cordless screwdriver in an initial overhead activity can be registered by logging in via an RFID tag at the workstation. This adjusts to the support curve intended for the activity with support over the entire angular range (corresponding to Figure 7A). The exoskeleton then compensates for the tool weight, and the user is now able to vary the support height individually and as needed via the rotary encoder on the control element, thereby tailoring it to him/herself and the context. The successful completion of the activity and the subsequent change to, for example, handling the wiring harness, is repeatedly detected by logging the RFID tag in and out. Thus, the support curve adjusts to the static support of overhead work (Figure 7B). The angular range to be supported in this process is limited to overhead activities, referenced to the horizontal, and monitored via the integrated IMU. The Lucy exoskeleton thus supports overhead activities in a standing position. If the user subsequently picks up a screw from the floor, this pre-defined scenario can be detected based on the bent position and support that would otherwise interfere with the activity can be prevented. Accordingly, support can also be designed based on whether the arm is moving up or down (Figure 7C). The adjustment of the support curve can be done by the perception of the environment and pre-defined scenarios as shown, or by the active selection of the user.

Through the bundled use of the connection and adaptation possibilities described above, it is possible to describe an exoskeleton that is adaptable to both the user and the situation and is even adaptive to a large extent concerning the support context.

## 5 Discussion

The framework enables a description of every possible support function. It offers the potential to describe existing support characteristics of exoskeletons and to derive and define future relevant system behavior. But even though the framework has been tested for exemplary prototypical active exoskeletons as described in the practical application example, it is paramount to consider occurring limitations or requirements. Generally, the framework is applicable for different actuation principles of exoskeletons, but the degree of suitability differs according to exoskeleton type. For example, its applicability fundamentally depends on exoskeletons’ installed sensor and actuator technology. Thus, the framework is to a greater extent geared towards active exoskeletons since, in the case of these, both a hardware- and software-technical adaptation of the support performance is possible. For the framework to not merely remain a theorized construct that cannot practically be implemented, it becomes apparent that the practicality of the framework in terms of adaptivity also needs further proof for commercially available exoskeletons. The current work situation with humans as the central actor in the industry, the remaining share of manual work activities, and market-available exoskeletons allow for further investigations in this regard. Here, however, the initiative of the exoskeleton manufacturers, in particular, is required since both software-based adaptation (e.g., in the form of sensor-based control for active exoskeletons) and hardware-based adaptation (e.g., in the form of additional coupling elements for the extension of the supported angular range or actuator technology with changed support characteristics) for commercial exoskeletons can usually only be performed by the respective suppliers themselves. However, due to its general formulation, no major obstacle is seen in applying the framework to commercial exoskeletons. In order to be able to implement a software-based adaptation according to the framework by allowing an adaptive control of the system behavior, exoskeletons must be capable of transmitting data and recording sensor values. In this regard, using artificial intelligence for data mining and neural networks in relation to the generated sensor data may help provide adaptive support for the respective application context. Besides, using sensor data from the exoskeleton offers the additional option of evaluating the user’s movements *in-situ* concerning ergonomics. At this point, a link with standard ergonomics methods is conceivable, which enables a live evaluation of body postures and movements. The use of neural networks can also help the exoskeleton realize and train an adaptive system behavior according to the requirements made by the context.

It is also crucial for which purpose and with which perspective the framework should primarily serve. Furthermore, it is crucial to define how the necessity and suitability of an adaptation of the support functions of exoskeletons can be measured. Following on from the description of the example, this is conceivable both in terms of anthropometric adaptation and adaptation of the support characteristics. Thus, there would be a great added value in not only naming the possible triggers of an adaptation but also in specifying them with regard to meaningful referencing points in time. Here, too, the use of artificial intelligence offers potential. When using the framework for determining the support functions of exoskeletons, it is always important to consider the intention and scope and whether support functions should be derived and defined holistically for entire activities or relate specifically to partial aspects such as individual movement segments. In connection with the support characteristics, it is still necessary to determine when the support should start and how dynamic adjustment can take place in support situations–not only in the amount and the course of the support but also whether support is required in the respective situation or not. A challenge in this context is identifying activities that trigger a need for support.

Nevertheless, the presented framework approach also offers further potential that can significantly impact the expansion of adaptive system behavior as well as the role of support technologies in future work in the industry. Thus, the interconnection of system technology offers the possibility of already taking the exoskeleton into account as an additional parameter in work preparation and production planning. For particular application contexts, the support functions can be adapted precisely to the movement, for example, on a methods time measurement (MTM) basis. In this regard, the use of sensors also makes it conceivable to identify possible subsequent processing steps in the work process using situation and intention recognition and to align the support function with the support context in a targeted manner (see the scenario described for RFID in the practical application). As a result, the support functions could be stored as presets and adaptively adjusted to the corresponding application context, and consequently, the appropriate support function can be selected. Thus, to a certain extent, required qualifications or even possible pre-existing conditions of employees can also be compensated by the support characteristics of the exoskeleton. In general, it is important to investigate how increasing adaptivity will affect the acceptance and usability of exoskeletons.

## 6 Conclusion

Given the prevalence and scope of musculoskeletal disorders in the workplace, exoskeletons are a promising support technology enabling workers to be supported during labor-intensive workplace stresses (such as performing repetitive tasks, working in constrained postures, or manipulating heavy loads). Concerning the support of the respective user, exoskeletons offer the possibility of providing targeted support in manual work processes due to their ability of user-specific and context-dependent adaptivity. In order to be able to reduce the strain on the workers and cope with the variety of complex requirements and conditions at workplaces, physical support appropriate to the situation is required. Adaptable and adaptive support systems represent a future-oriented way of achieving this (see chapter 2 for a more detailed answer to RQ1). Accordingly, the adaptive system behavior of exoskeletons plays a crucial role in this process. Against this background, a framework has been described enabling the classification of exoskeleton’s support characteristics according to a standardized scheme (see chapter 3 for a more detailed answer to RQ2). Following a modular principle, the support functions of exoskeletons can thus be described in more detail. By further qualifying exoskeletons in terms of adaptability, users can individually and contextually experience the support that helps them most in the given situation. Even though a concrete application of the framework is described as a procedure and for a practical scenario (see chapter 4 for a more detailed answer to RQ3), it mainly remains abstract. It requires further tests by extending the exemplary application to other exoskeletons. Nevertheless, the framework represents a novel and holistic approach to describing the support characteristics of physical support technologies such as exoskeletons.

## Data Availability

The original contributions presented in the study are included in the article, further inquiries can be directed to the corresponding author.
